# Supplementary material for: Octupole moment driven free charge generation in partially chlorinated subphthalocyanine for planar heterojunction organic photodetectors
Source: Nat Commun. 2024 Jun 13;15:5058. doi: 10.1038/s41467-024-49169-1 (PMC11176190; doi:10.1038/s41467-024-49169-1)
Supplement: Supplementary file 1 — Supplementary Information [file 41467_2024_49169_MOESM1_ESM.pdf]

Supplementary Information

Octupole moment driven free charge generation in partially chlorinated subphthalocyanine for planar heterojunction organic photodetectors

Aniket Rana[1,8], Song Yi Park[2,6,8], Chiara Labanti[2,8], Feifei Fang[3,8], Sungyoung Yun[3], Yifan Dong[1,7], Emily J. Yang[2], Davide Nodari[1], Nicola Gasparini[1], Jeong-Il Park[3], Jisoo Shin[3], Daiki Minami[4], Kyung-Bae Park[3,*], Ji-Seon Kim[2,*], James R. Durrant[1,5,*]

[1]Department of Chemistry and Centre for Processable Electronics, Imperial College London, London W12 0BZ, UK

[2]Department of Physics and Centre for Processable Electronics, Imperial College London, London SW7 2AZ, UK

[3]Organic Materials Lab, Samsung Advanced Institute of Technology, Samsung Electronics Co. Ltd., Samsung-ro, Yeongtong-gu, Suwon-si, Gyeonggi-do, 16678, Republic of Korea

[4]Innovation Center, Samsung Electronics, Co. Ltd., 1 Samsungjeonja-ro, Hwaseong-si, Gyeonggi-do, 18448, Republic of Korea

[5]SPECIFIC, Faculty of Science and Engineering, Swansea University, Swansea, SA1 8EN, UK

[6]Present address: Department of Physics, Pukyong National University, Busan 48513, Republic of Korea

[7]Present address: National Renewable Energy Laboratory, 15013 Denver W Pkwy, Golden, CO 80401, United States

[8]These authors contributed equally: Aniket Rana, Song Yi Park, Chiara Labanti, Feifei Fang

[*]Corresponding Author:

Kyung-Bae Park (myshkin.park@samsung.com), Ji-Seon Kim (ji-seon.kim@imperial.ac.uk), James R. Durrant (j.durrant@imperial.ac.uk).

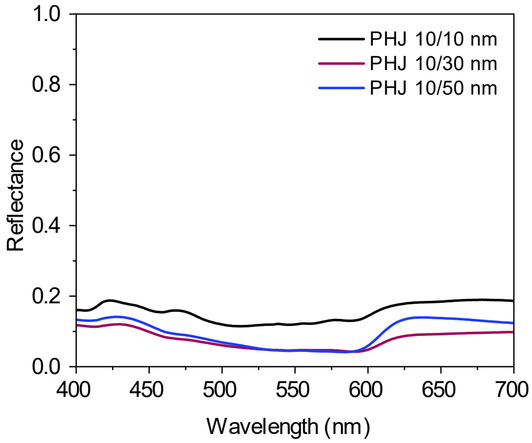

Supplementary Fig. 1. PHJ OPD device reflectance. Relative reflectance spectra normalized against Barium Sulfate as reflective control for PHJ 10/10 nm, PHJ 10/30 nm and PHJ 10/50 nm device stacks.

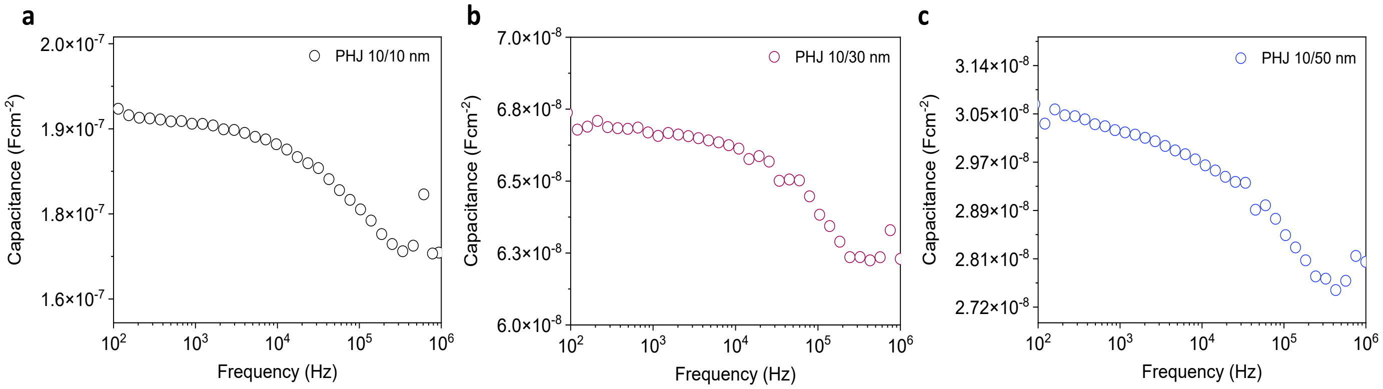

Supplementary Fig. 2. Capacitance response with respect to frequency under dark for trap density analysis. **a** PHJ 10/10 nm **b** PHJ 10/30 nm **c** PHJ 10/50 nm devices.

The calculation of trap density (tDoS) utilizes capacitance frequency measurement which corresponds to the fraction of thermally excited trapped charges responding to the applied AC field. Supplementary Fig. S1 shows the capacitance response for all PHJ OPD devices. The applied AC field alternately traps and release carriers which are present near to the Fermi energy. The trap energy related to the measurement frequency by the following equation (1)[1]

$$E_t = kT\ln\left(\frac{\omega_0}{\omega}\right) \tag{1}$$

Where k is Boltzmann's constant, T is the temperature, and ω_0 is rate perfector for thermal excitation of carriers from trap and typically for organic photodiode, it is 10^{12} Hz and ω is AC perturbation frequency[2,3]. The technique for detecting traps states from the capacitive response was originally proposed by Walter et. al[4] and given by equation (2)

$$\mathrm{tDoS}(E_t) = -\frac{V_{bi}}{qW}\frac{dC}{d\omega}\frac{\omega}{kT} \tag{2}$$

where q is electric charge, C is measured capacitance with respect to frequency, V_{bi} is built in potential, W is thickness of PHJ layer. The change in slope $dC/d\omega$ reflects the increase of trap density with respect to the trap energy. To estimate the σ (disorder parameter) and mean trap energy (E_0) trap density is fitted with Gaussian distribution function as given in equation (3)

$$\mathrm{tDoS}(E_t) = \frac{\mathrm{tDoS}}{\sqrt{2\pi}\sigma}\exp\left[\frac{-(E_0-E_t)^2}{2\sigma^2}\right] \tag{3}$$

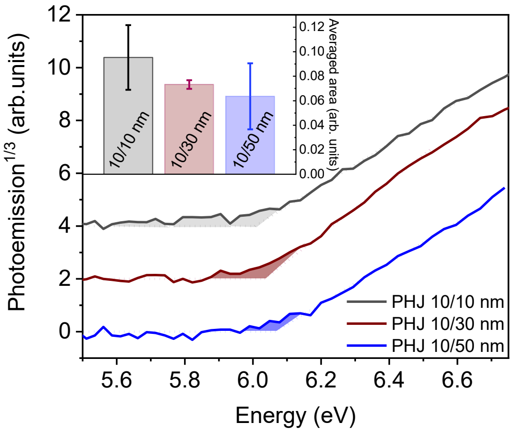

Supplementary Fig. 3. Sub-bandgap tail states analysis. The photoemission spectra for PHJ 10/10 nm, PHJ 10/30 nm and PHJ 10/50 nm devices. Shaded area illustrates additional overall trap states in devices. Inset shows integrated area of these trap states which also qualitatively indicates that PHJ 10/50 nm has least trap states. Error bars are shown as standard deviation in a sample of several repeated experimental measurements.

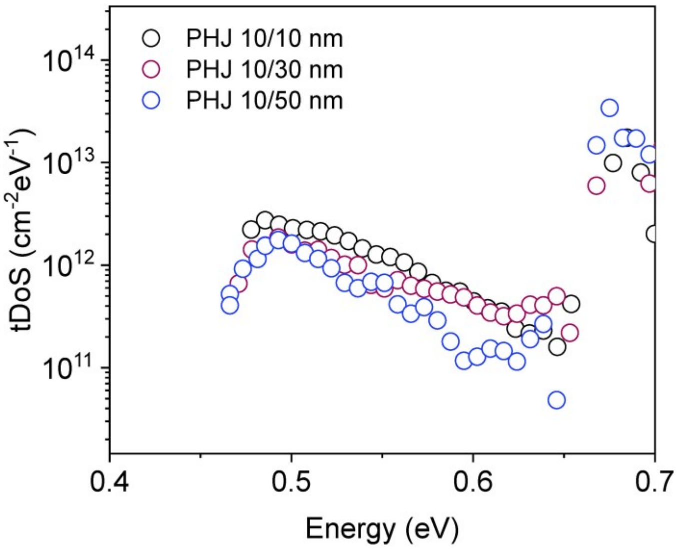

Supplementary Fig. 4. Interfacial traps density. Trap density distribution per unit area determined from capacitance frequency measurements under dark condition to estimate interfacial traps.

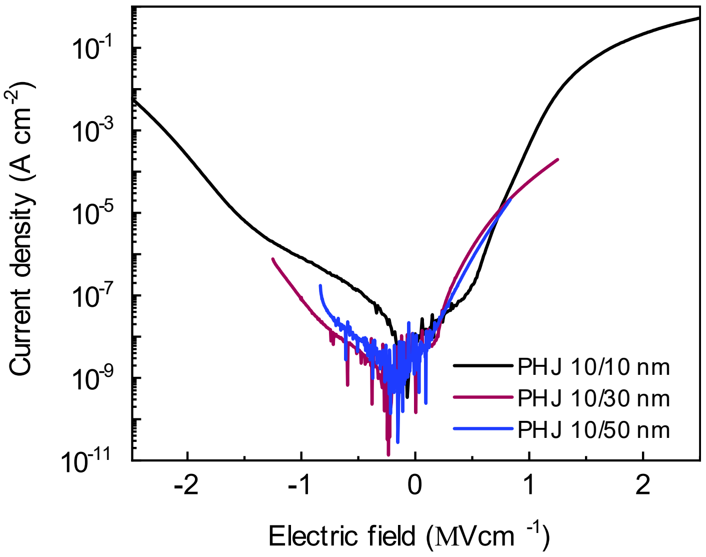

Supplementary Fig. 5. Electric field dependent PHJ OPD response under dark condition. Current density with respect to the electric field characteristics of PHJ MPTA/Cl_6-SubPc photodiodes for different thicknesses of acceptor under dark conditions.

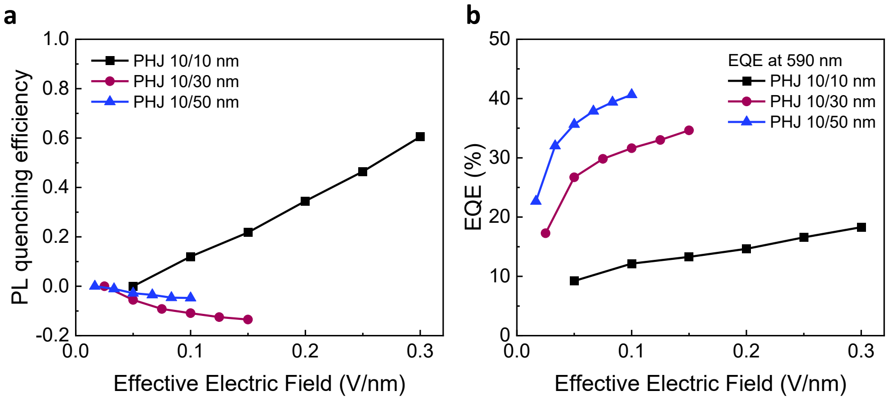

Supplementary Fig. 6. Field dependent PL quenching and quantum efficiency. a PL quenching efficiency of PHJ devices as a function of the effective electric field within overall wavelengths. **b** Summary of EQE increase as a function effective electric field.

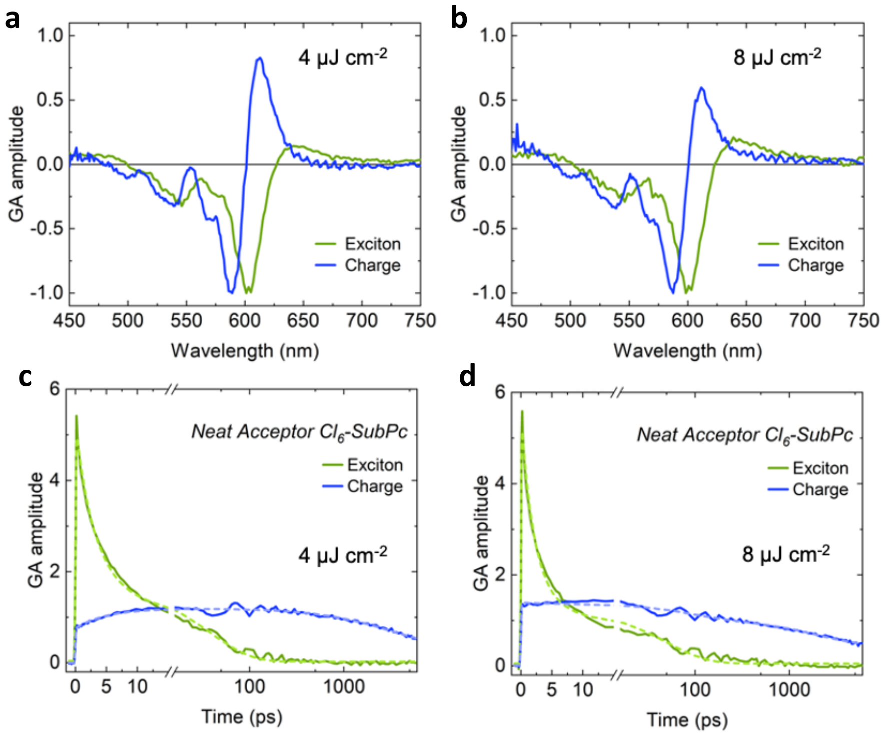

Supplementary Fig. 7. Excitation fluence dependent charge carrier generation. Transient absorption spectra after global analysis at excitation fluences **a** 4 μJcm^{-2} **b** 8 μJcm^{-2} and

corresponding kinetics for excitons and charges at **c** 4 μJcm^{-2} **d** 8 μJcm^{-2} excitation fluences for neat Cl_6-SubPc films.

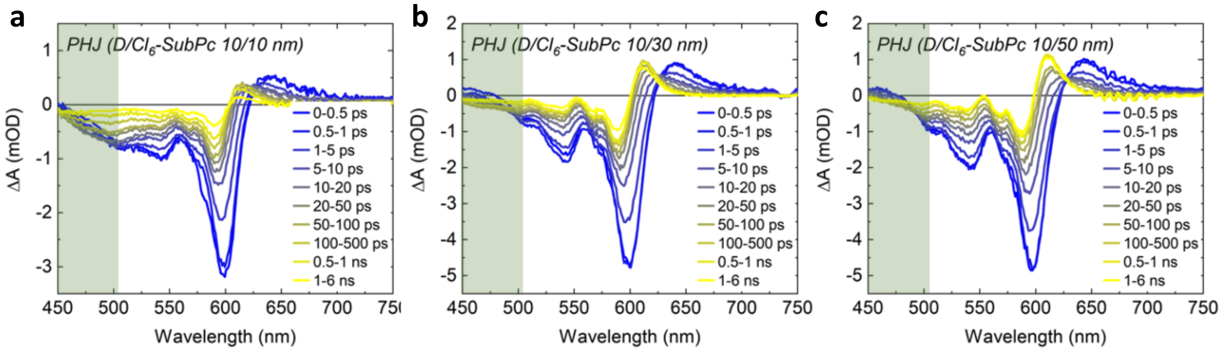

Supplementary Fig. 8. Transient absorption spectra for PHJ MPTA/Cl_6-SubPc films with varying acceptor thicknesses. For **a** PHJ 10/10nm **b** PHJ 10/30nm **c** PHJ 10/50nm.

Samples were excited at 610 nm, selectively exciting Cl_6-SubPc, with a fluence of 6 μJ cm^{-2}. Data for 10/30 nm and 10/50 nm bilayers are almost indistinguishable from neat Cl_6-SubPc. The thinnest 10/10 nm bilayer is also very similar, with only a small additional bleach signal around 500 nm, most likely resulting from minor MPTA photoexcitation.

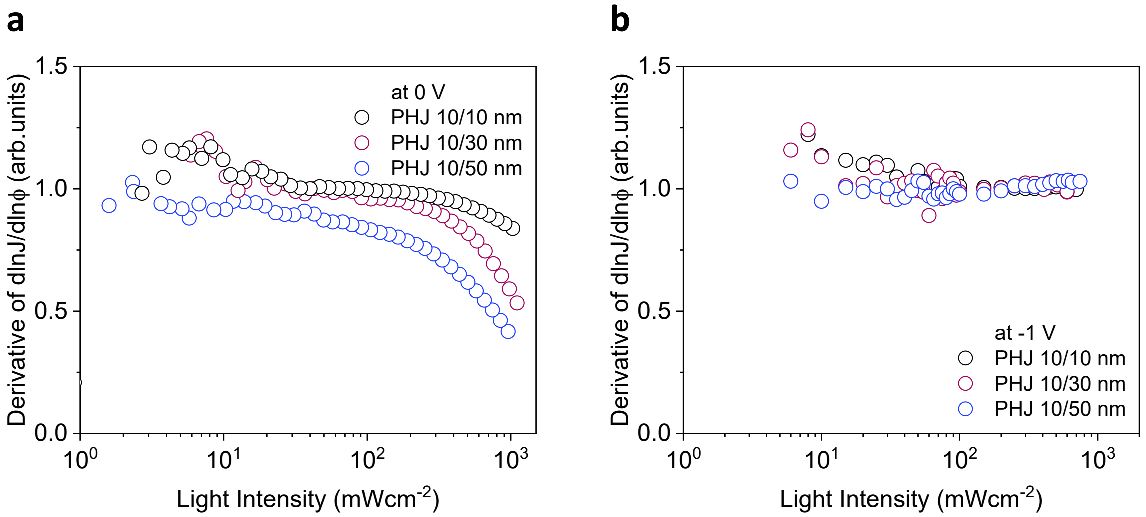

Supplementary Fig. 9. Photo current linearity. Derivative of ln(J_{SC}) as function of light intensity under **a** 0 V (no bias) **b** -1 V

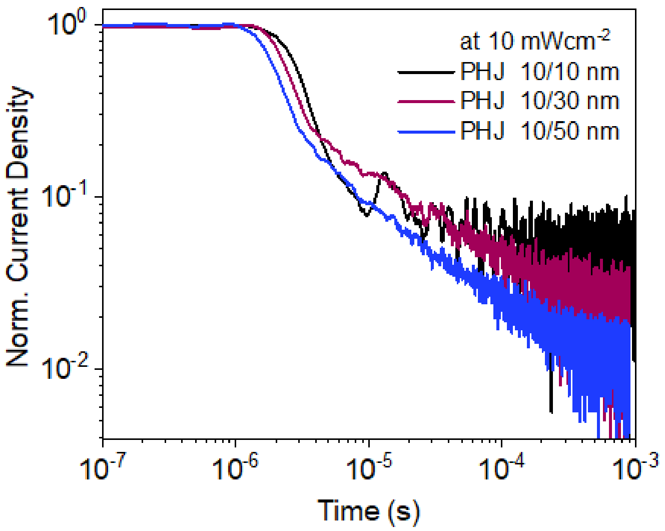

Supplementary Fig. 10. Device photocurrent transient response. Measured by charge extraction (CE) at 0 V and under 10 mWcm^{-2} white light. PHJ 10/10 nm shows high current (slow decay phase) due to the highest trap density as compared to the thick devices.

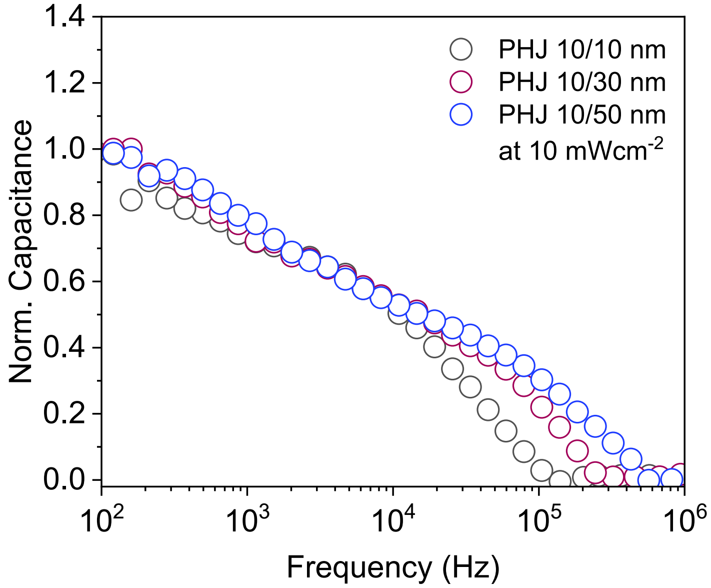

Supplementary Fig. 11. PHJ OPD frequency dependent capacitance response under light. Normalized capacitance response with respect to frequency at 10 mWcm^{-2} white light intensity indicating capacitance drop shift to high frequency in with increase in Cl_6-SubPc thickness.

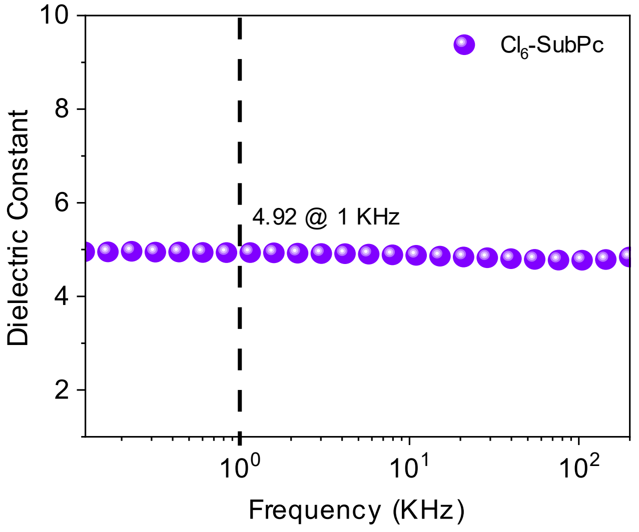

Supplementary Fig. 12. Dielectric analysis of Cl_6-SubPC film. Dielectric constant with respect to frequency ranging from 0.1 KHz to 200 KHz for single layer Cl_6-SubPc (50nm) without MPTA. At frequency of 1 KHz, the recorded dielectric constant is 4.92[5].

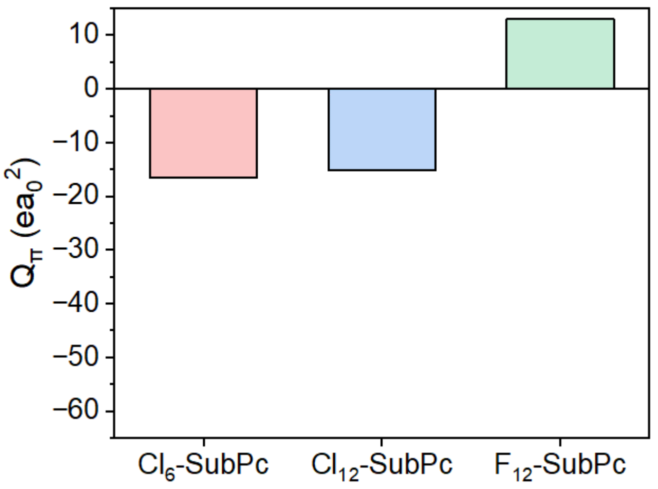

Supplementary Fig. 13. Quadruple moment comparison. Single molecule DFT calculations for quadruple moment in different SubPc derivatives.

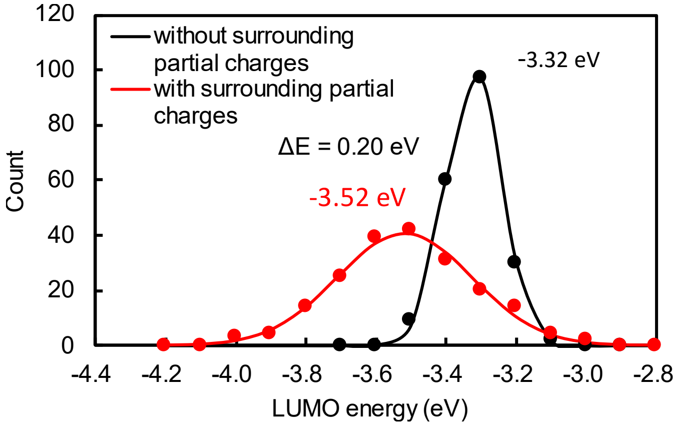

Supplementary Fig. 14. Energy level distributions from MD simulation. LUMO of Cl_6-SubPc's in each cluster calculated without/with inclusion of the partial charges associated with Cl_6-SubPc's high octupole moment.

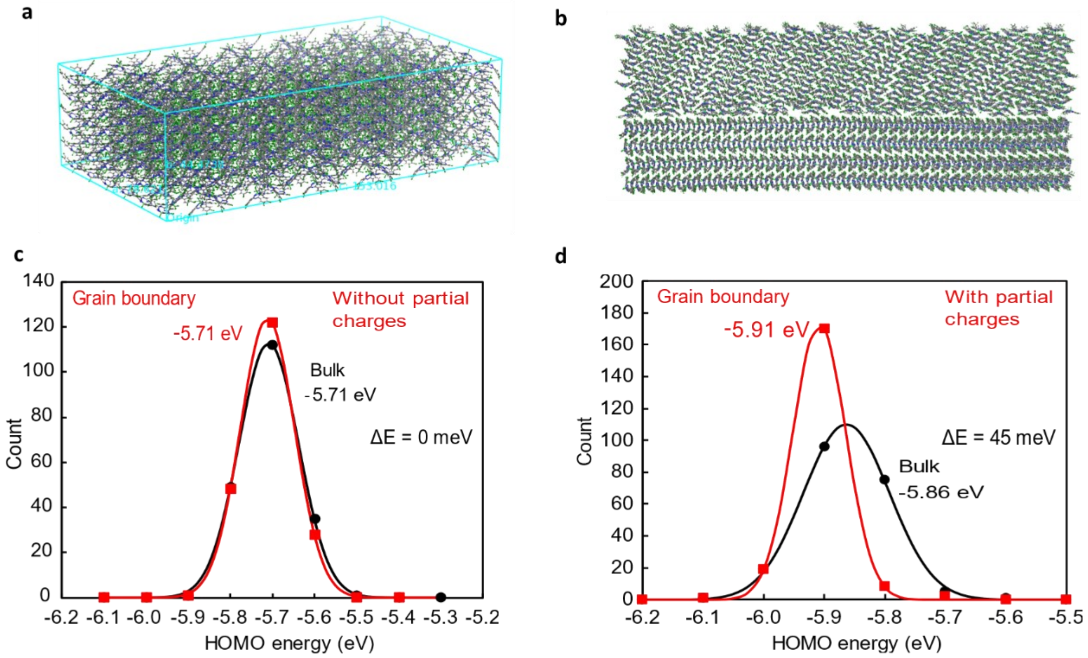

Supplementary Fig. 15. MD simulations of film structures and energetics. **a** The crystal structure of 1000 molecules of Cl_6-SubPc generated from molecular dynamics simulations. **b** The grain boundary morphology obtained by such molecular dynamic simulations **c** HOMO energy level distributions of randomly selected clusters of Cl_6-SubPc molecules in the bulk or at grain

boundaries without inclusion of the partial charges associated with Cl_6-SubPc's high octupole moment. **d** HOMO energy distributions as for **c** but with inclusion of the partial chargers associated with Cl_6-SubPc's high octupole moment.

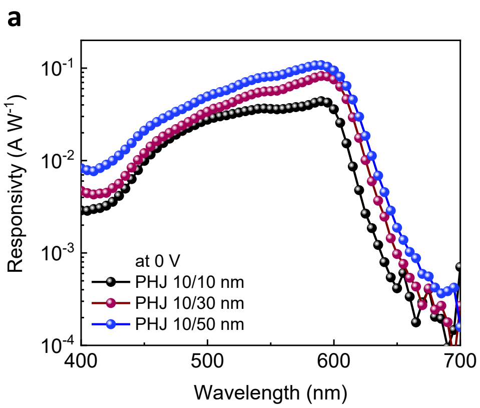

Supplementary Fig. 16. **Thickness dependent photodetector spectral response.** Spectral responsivity versus wavelength under different bias calculated from EQE at **a** 0V and **b** -1V for all PHJ OPDs.

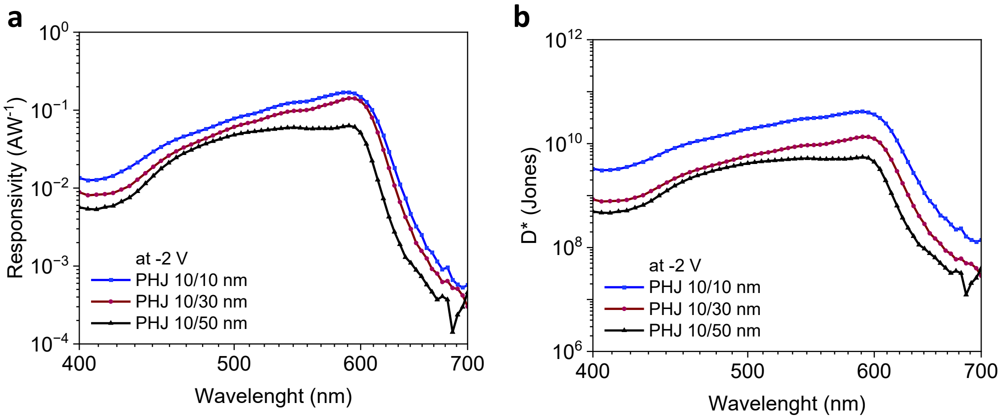

Supplementary Fig. 17. Thickness dependent photodetector performance parameter under -2V bias. **a** Spectral responsivity versus wavelength and **b** specific detectivity (D^*_{noise}) versus wavelength calculated from EQE at -2V for all PHJ OPDs by considering noise spectral density (see Supplementary Fig. 18).

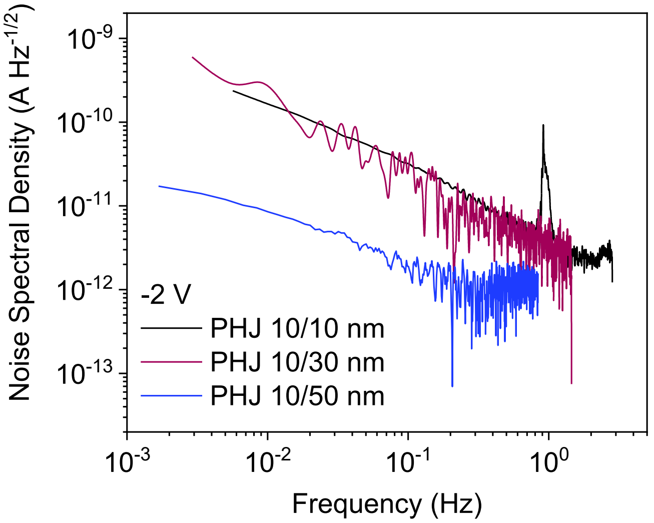

Supplementary Fig. 18. Noise measurements. Spectral noise density at -2 V for all OPD devices PHJ 10/10nm, PHJ 10/30 nm and PHJ 10/50 nm.

The noise referenced specific detectivity (D^*_{noise}) spectra at a reverse bias of -2 V were calculated using $D^*_{noise}=R\sqrt{A\Delta f}/i_n$, where R is the responsivity, A is the active area, Δf is the detection bandwidth and i_n is the noise current measured as described in the methods section and depicted in Supplementary Fig. S18. i_n can also be modelled by the following equation (4).

$$(i_n)^2 = \left(2qi_d + \frac{4kT}{R_{sh}} + i_{1/f}^2\right)\Delta f \tag{4}$$

where q is the elementary charge, i_d is the dark current, k is the Boltzmann constant, T is the temperature, R_{sh} is the shunt resistance and $[i_{1/f}]^2$ is the flicker noise spectral density[6,7]. The detectivity data reported in the main text are calculated just considering $2qi_d$ (shot noise) as main contribution to the overall noise of the devices.

Supplementary References

1. Rana, A., Kumar, A., Chand, S. & Singh, R. K. Exploring deep defect state impact on open circuit voltage of conventional and inverted organic solar cells. *J Appl Phys* **124**, 103101 (2018).

2. Yao, W. *et al.* Organic Bulk Heterojunction Infrared Photodiodes for Imaging Out to 1300 nm. *ACS Appl Electron Mater* **1**, 660–666 (2019).

3. Kublitski, J. *et al.* Reverse dark current in organic photodetectors and the major role of traps as source of noise. *Nat Commun* **12**, 551 (2021).

4. Walter, T., Herberholz, R., Müller, C. & Schock, H. W. Determination of defect distributions from admittance measurements and application to Cu(In,Ga)Se_2 based heterojunctions. *J Appl Phys* **80**, 4411–4420 (1996).

5. Hughes, M. P. *et al.* Determining the Dielectric Constants of Organic Photovoltaic Materials Using Impedance Spectroscopy. *Adv Funct Mater* **28**, 1801542 (2018).

6. Fang, Y., Armin, A., Meredith, P. & Huang, J. Accurate characterization of next-generation thin-film photodetectors. *Nature Photon* **13**, 1–4 (2019).

7. Ho Lee, T. *et al.* Organic Planar Heterojunction Solar Cells and Photodetectors Tailored to the Exciton Diffusion Length Scale of a Non-Fullerene Acceptor. *Adv Funct Mater* **32**, 2208001 (2022).
